# Carotid Doppler Imaging as a Marker for Fluid Responsiveness

**DOI:** 10.3390/jcm14186657

**Published:** 2025-09-22

**Authors:** Ankur Srivastava, Christopher Tam, Samir Sethi, Mario Gaudino, Brady Rippon, Joydeep Baidya, Sanya Rastogi, Alexandra Lopes, Avika Kasubhai, Kane Pryor, James Osorio

**Affiliations:** 1 Department of Anesthesiology, New York-Presbyterian Hospital/Weill Cornell Medicine, New York, NY 10065, USA; ctam@motefiore.org (C.T.); ssethi@aam-nj.com (S.S.); jbortho98@gmail.com (J.B.); sr1855@njms.rutgers.edu (S.R.); alex.lopes@ucsf.edu (A.L.); avikakasubhai@gmail.com (A.K.); kap9009@med.cornell.edu (K.P.); 2 Department of Anesthesiology, Montefiore Medical Center, Bronx, NY 10467, USA; 3 Anesthesia Associates of Morristown, Morristown, NJ 07960, USA; 4 Department of Cardiothoracic Surgery, New York-Presbyterian Hospital/Weill Cornell Medicine, New York, NY 10065, USA; mfg9004@med.cornell.edu; 5 Department of Population Health Sciences, Division of Biostatistics, Weill Cornell Medicine, New York, NY 10065, USA; brr7014@med.cornell.edu; 6 College of Medicine, Downstate Health Sciences University, Brooklyn, NY 11203, USA; 7 Rutgers New Jersey Medical School, Newark, NY 07103, USA; 8 School of Medicine, University of California, San Francisco, CA 94143, USA; 9 School of Medicine, Georgetown University, Washington, DC 20007, USA

**Keywords:** fluid responsiveness, central venous pressure (CV), carotid Doppler ultrasound, point-of-care-ultrasound (POCUS), pulmonary artery catheter (PAC), cardiac output (CO), passive leg raise, corrected flow time (FTc), respiratory peak carotid systolic velocity (ΔCDPV), carotid blood flow

## Abstract

**Background/Objective:** Identifying fluid-responsive patients is essential in managing hemodynamic instability. Traditional static measures like central venous pressure (CVP) are often unreliable. Prior studies suggest that cardiac ultrasound (US), particularly carotid Doppler point-of-care ultrasound (POCUS), may correlate with pulmonary artery catheter (PAC)-derived cardiac output (CO), offering a noninvasive tool to assess fluid responsiveness. We aimed to evaluate the correlation between carotid ultrasound (US) parameters and pulmonary artery catheter (PAC) derived measurements in post cardiac surgery patients. **Methods:** We conducted a prospective cohort study on 50 postcardiac surgery patients from 2019 to 2022 in a single cardiothoracic ICU. Carotid US and PAC CO measurements were obtained at four intervals: pre- and post-passive leg raise (fluid challenge) on ICU admission, and one hour later. Fluid responsiveness was defined as a ≥10% increase in carotid blood flow, ≥7 ms increase in corrected flow time (FTc), or ≥10% change in respiratory peak carotid systolic velocity (ΔCDPV). Pearson’s correlation and linear regression were used to assess associations between carotid US and PAC changes. Agreement in fluid responsiveness categorization (≥10% CO change) was evaluated using weighted Cohen’s kappa. Significance was set at α = 0.05. **Results:** No significant correlation was found between changes in carotid US parameters and the PAC cardiac index (CI) at baseline or one hour for ΔCDPV, FTc, or carotid blood flow. A moderate correlation was observed between carotid blood flow and FTc at one hour (r = 0.41, *p* = 0.005). Regression and sensitivity analyses showed no significant associations. **Conclusions:** The carotid US parameters did not correlate with PAC-derived CO after passive leg raise. Further studies are needed to validate carotid POCUS in this setting.

## 1. Introduction

There are many ways to assess volume status in critical care settings and, therefore, determine the need to administer fluid as therapy. This includes a detailed history and physical exam, laboratory data, markers of renal function, and radiographic imaging, all invaluable and usually readily available.

Fluid responsiveness is defined as an increase in stroke volume and, therefore, cardiac output by approximately 10–15% after fluid administration [1,2,3,4]. This is important information that aids in the management of critically ill patients. Not all hypotensive patients or patients in shock are fluid responsive, and administering additional empiric fluid boluses carries the risk of adding more fluid to patients already functioning on the steep portion of their Frank–Starling curve. This, in turn, could be manifested as fluid overload, with associated increased morbidity and worse outcomes [5]. The means to accurately determine stroke volume, cardiac output, and fluid responsiveness are thus of paramount clinical significance. 

In clinical practice, the pulmonary artery catheter (PAC) is widely regarded as the gold standard for measuring cardiac output, having been the first such monitor introduced in the 1970s. At the time, PAC measurements demonstrated reasonable correlation with the Fick method, typically within a 10–20% error margin. However, the PAC was adopted into routine use without undergoing the rigorous evaluation now expected of more recently developed, less invasive cardiac output monitors.

The Fick method, while theoretically more accurate, is cumbersome and impractical for clinical practice—particularly due to the challenges of measuring oxygen consumption. Bland–Altman analysis was introduced in 1986 as the preferred method for evaluating agreement between measurement techniques.

Newer technologies are typically validated against the PAC using Bland–Altman analysis, which assesses bias, limits of agreement (LOAs), and standard deviation. For regulatory approval, devices must demonstrate agreement within two standard deviations of PAC values—a threshold that still permits notable variability, especially under complex clinical conditions, such as shock states, arrhythmias, and scenarios involving rapid shifts in blood volume and blood density.

There are several less-invasive and noninvasive methods for estimating stroke volume and cardiac output [6,7]. Among them is the validated Doppler method used in point-of-care ultrasound (POCUS), readily available at the bedside [8].

As an alternative to visualizing the pulsed wave Doppler in the left ventricular outflow tract (LVOT VTI) or assessing collapsibility of the superior or inferior vena cava (SVC/IVC) during cardiac POCUS, evaluation of pulsed wave Doppler signals in the common carotid artery has been proposed to assess volume responsiveness. The common carotid artery is much easier to identify anatomically and is in a more accessible location, especially in unstable patients under the direct care of anesthesiologists, critical care, and emergency medicine physicians [9].

Each available method—whether based on ultrasound, contour analysis, thermodilution, indicator dilution, bioreactance, plethysmography, or end-expiratory occlusion—has known limitations. To help mitigate heart–lung interactions associated with positive pressure ventilation in mechanically ventilated patients, the passive leg raise (PLR) test combined with the selected measurement modality is commonly used [10].

Recent studies have explored the correlation between carotid artery ultrasound measurements and cardiac output-based assessments, particularly in comparison to noninvasive cardiac output monitors, such as NICOM (bioreactance-based noninvasive cardiac output monitor, Cheetah NICOM^®^ system, Boston, Newton, MA, USA) and pulmonary artery catheter measurements.

This relationship has been investigated in both ICU patients experiencing shock and in non-critically ill individuals undergoing cardiac catheterization [11]. Notably, respiratory peak systolic carotid velocity emerged as a predictor of volume responsiveness in mechanically ventilated patients with septic shock [12]. Additionally, the variation in carotid flow time (FTc) demonstrated a moderate correlation with cardiac output measured through thermodilution (Ma et al., Crit Ultrasound J. 2017) in these clinical scenarios [13]. These findings suggest the potential utility of carotid artery ultrasound in assessing hemodynamic status across diverse patient populations and critical care contexts, including postoperative cardiothoracic surgery patients [14,15,16,17].

Despite limitations, including the need for repeated measurements, equipment, and expertise, using ultrasound for estimating cardiac output remains an attractive option given its relative ubiquity nowadays in ICUs and ERs alike.

Our cardio–thoracic ICU (CTICU) cares for post-cardiac surgery patients, all of whom receive a PAC for monitoring in the operating room. Therefore, this study aimed to determine whether there is a correlation between carotid US and PAC CO measures in postoperative cardiac surgery patients.

We hypothesized that POCUS of the common carotid artery and the subsequent measurement of carotid blood flow (CBF), corrected carotid flow time (FTc) with respiration and passive leg raise, and carotid Doppler peak systolic velocity variability (∆CDPV) are accurate and reliable methods for assessing fluid responsiveness that will correlate with pulmonary artery catheter data.

∆CDPV, also referred to as variation in peak Doppler carotid velocity, is widely utilized in assessing peak-to-peak variability of the arterial waveform—a parameter used to predict fluid responsiveness (Ibarra-Estrada et al., Crit Ultrasound J.2015) [12].

## 2. Materials and Methods

### 2.1. Study Design, Population, and Setting

We conducted a single-center prospective cohort study from August 2020 to October 2021 at a large tertiary care hospital affiliated with an academic medical center. The Institutional Review Board at Weill Cornell Medical College approved this study. We obtained written consent from all participants.

### 2.2. Inclusion and Exclusion Criteria

**Figure 1:**
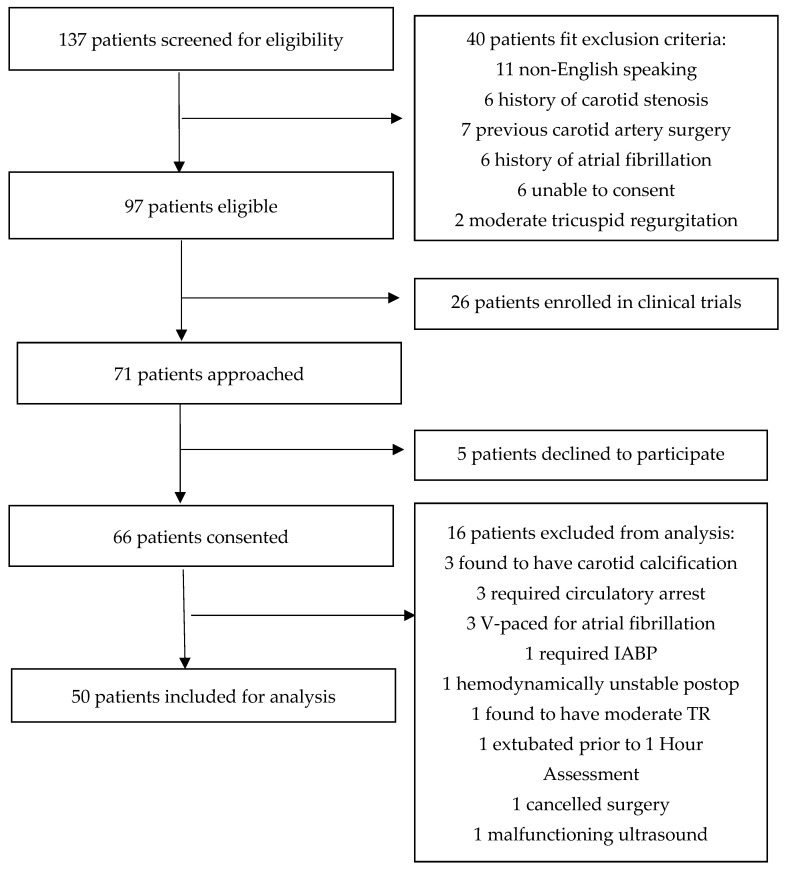
Patient screening and enrollment.

We included postoperative surgical patients aged above 20 years old, admitted postoperatively to the Critical Intensive Care Unit (CTICU), who had sinus rhythm or atrial pacing, were mechanically ventilated, and had an indwelling PAC. We excluded patients who did not meet these criteria, as well as those who had carotid stenosis greater than 50% (determined by preoperative ultrasound or previous carotid artery surgery), at least moderate tricuspid regurgitation, or intracardiac shunts noted on intraoperative TEE (Figure 1).

### 2.3. Measurements

**Scheme 1:**
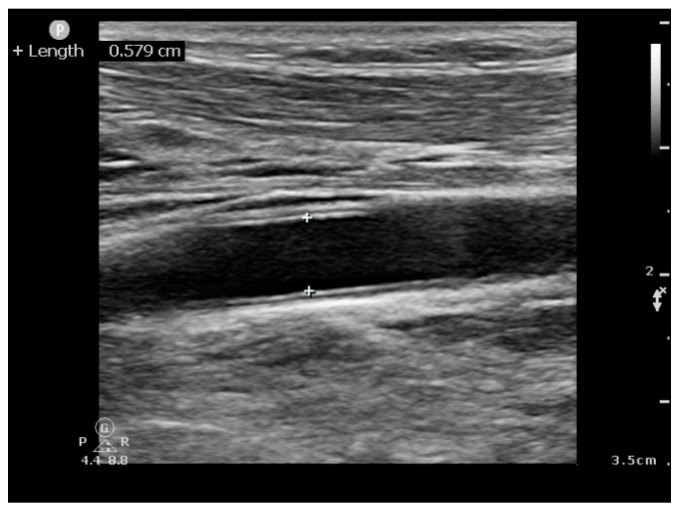
This is a longitudinal view of the common carotid artery (CCA) that was used to measure diameter and derive area.

**Scheme 2:**
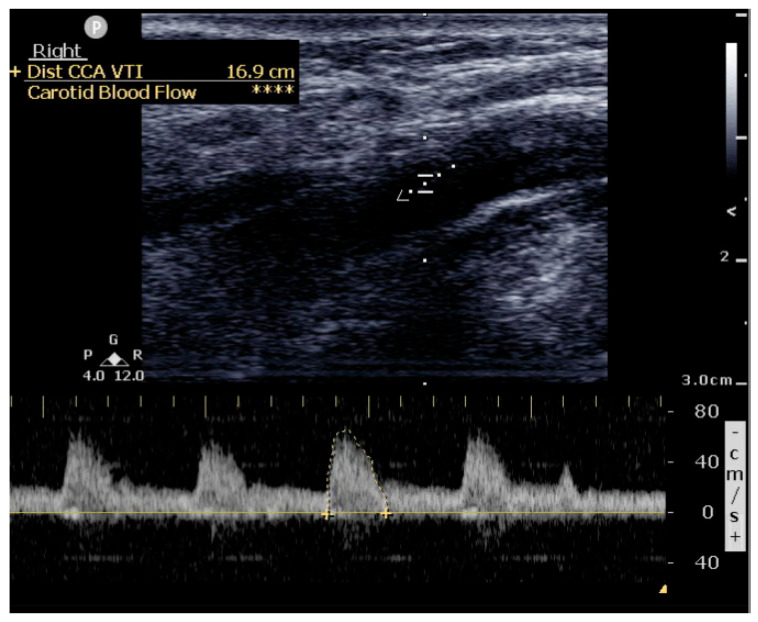
**** These are pulsed wave carotid doppler clips. For accuracy of measurements, angle of interrogation needs to be less than 60 degrees.

**Scheme 3:**
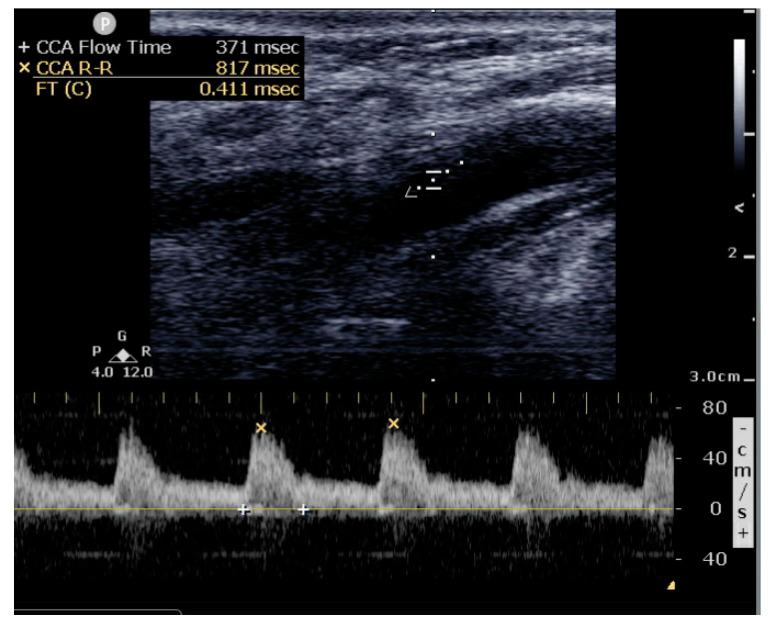
This is a pulse doppler view of the common carotid artery (CCA) that was used to measure systolic time (ST) and Cycle time (CT) which were used to calculate corrected Flow time (FTc).

Intensivists trained in ultrasonography used Philips SPARQ to acquire images with a linear probe 4–12 mHz. We performed carotid US and cardiac measures at four intervals: an initial assessment on patient ICU admission, pre- and post-passive leg-raise, as well as at one hour post initial assessment, pre- and post-passive leg-raise (Scheme 1, Scheme 2 and Scheme 3).

The rationale for selecting the immediate ICU admission period and one hour thereafter for our measurements is based on physiological considerations specific to the early postoperative phase following cardiac surgery. During this time, patients often experience relative hypovolemia due to early postoperative fluid shifts, making them more likely to be fluid responsive. This state can be reliably detected by observing increases in stroke volume and cardiac output during a passive leg raise maneuver, measured via pulmonary artery catheter (PAC). We also hypothesized that carotid Doppler measurements obtained through point-of-care ultrasound (POCUS)—including changes in stroke volume and corrected flow time (FTc)—would similarly reflect fluid responsiveness during this period.

We estimated the volume of blood ejected per cardiac cycle, or the stroke volume, by ultrasound by applying the following formula:

(SV = CCA PWD VTI × CCA Area).

CCA PWD VTI—Common Carotid Artery Pulsed Wave Doppler Time Velocity Integral.
CCA Area—Common Carotid Artery Area = D^2^ × 0.785.

The corrected flow time (FTc) reflects the systolic ejection time adjusted for heart rate, typically using Bazett’s formula. A longer FTc is generally associated with increased stroke volume and cardiac output.

**Systolic time** refers to the duration from the onset of the systolic upstroke on the carotid pulsed-wave Doppler signal to the return to baseline, marking the end of systolic ejection.

**Cycle time** is defined as the interval from the beginning of one carotid Doppler systolic upstroke to the beginning of the next, corresponding to one complete cardiac cycle.

The rationale for using FTc to assess fluid responsiveness is based on the expected prolongation of ejection (systolic) time following a fluid challenge in fluid-responsive patients. This method has been validated in several studies and is supported by the availability of bedside monitors, including point-of-care ultrasound (PoCUS).

In this study, FTc was estimated using carotid Doppler ultrasound and calculated with Bazett’s formula [17].

FTc = ST/√CT, where ST is systolic time and CT, cycle time.

We measured a change in ultrasound peak carotid pulsed wave Doppler velocity during respiratory variation, and we performed the calculation using the following formula:

∆Peak Carotid Velocity = [(Max − Min)/((Max + Min)/2)] × 100

We defined fluid responsiveness by carotid POCUS using the following 3 elements:

1. Increase in carotid blood flow by 10% after a fluid challenge. (Passive leg raise).
2. 7 msec increase in corrected flow time (FTc) after fluid challenge.
3. 10% change in peak carotid systolic velocity (ΔCDPV).

### 2.4. Statistical Analysis

Since this is an exploratory pilot study, a power calculation is not required.

First, we evaluated associations between changes in carotid measures and changes in cardiac PAC measures with the use of Pearson’s correlation coefficients. We further tested these associations through linear regression models, both unadjusted and adjusted for demographics. We further analyzed carotid and cardiac PAC measures by categorizing changes from pre- to post-passive leg raise as: (1) decreased by 10%; (2) increased by 10%; or (3) no meaningful change (less than 10% change in either direction). We evaluated agreement between carotid and cardiac classifications using a weighted Cohen’s kappa. We also investigated different cutoffs for meaningful change in a sensitivity analysis, with agreement by weighted Cohen’s kappa considered as an optimization parameter. We reported results at an α = 0.05 significance level. We performed the analysis using R v4.3.0.

## 3. Results

### 3.1. Description of Study Sample

In this sample of 50 subjects, most patients were male (76%). Most patients had a history of hypertension cases (58%), half had abnormal HDL (50%), one-third had coronary artery disease cases, and 16% of patients had diabetes mellitus (Table 1).

### 3.2. Correlation Analysis

**Figure 2:**
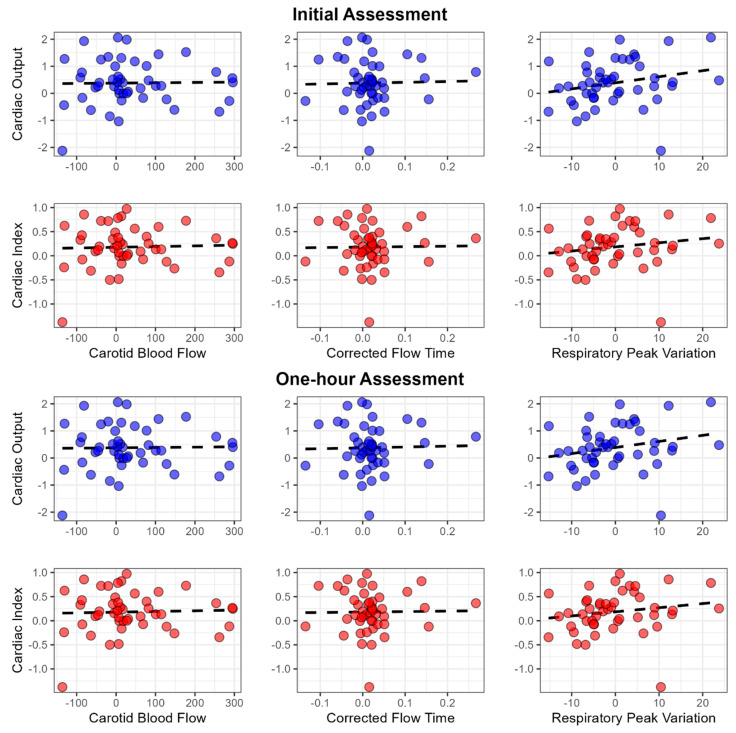
Scatterplots comparing the change in carotid measures (columns) to the change in PAC cardiac output measures (rows). Each column contains the change from pre- to post-passive leg raise for carotid blood flow, corrected flow time, and respiratory peak variation, respectively, within the appropriate assessment window. The dashed line in each plot represents the linear trend between the measures.

**Figure 3:**
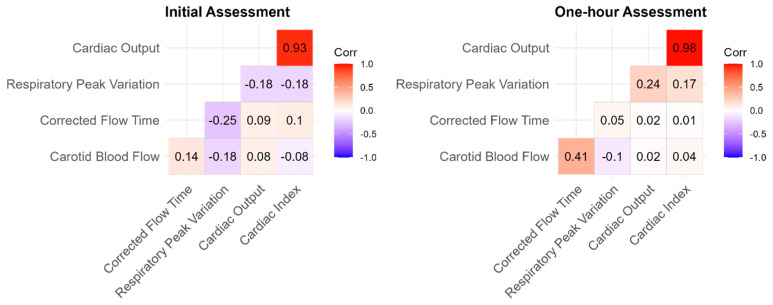
Pearson’s correlations comparing the change from pre- to post-passive leg raise in carotid measures and the change in PAC cardiac output measures.

First, we used scatterplots and Pearson’s correlation coefficients to examine the strength, direction, and form of the relationship between the change in carotid US (cardiac index) to PAC cardiac output measures, as the change from pre- to post-passive leg raise for carotid blood flow, corrected flow time, and respiratory peak variation, respectively (Figure 2 and Figure 3). We did not detect significant correlations between any change in carotid measures and any change in PAC cardiac output measures for either assessment window.

### 3.3. Linear Regression Modeling

Next, we used linear regression modeling (unadjusted and adjusted for sex, body surface area, and disease history) to assess associations between a change in carotid US measures (cardiac index) and a change in PAC cardiac output measures from pre- to post-passive leg raise. We did not detect significant associations in linear regression models between any change in carotid measures and any change in PAC cardiac output measures for either assessment window (Table 2).

### 3.4. Sensitivity Analysis

**Figure 4:**
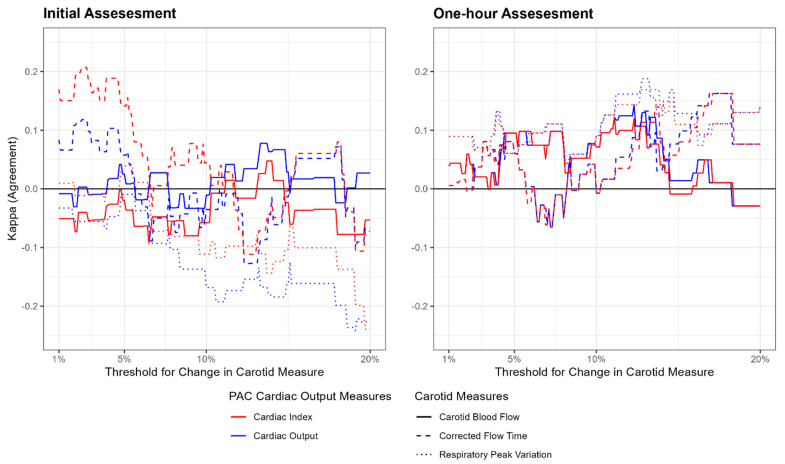
Weighted Cohen’s Kappa for the agreement between categorical changes from pre- to post-passive leg raise in carotid measures and PAC cardiac output measures. The x-axis represents a spectrum of cutoffs for meaningful change in carotid measures, while the y-axis represents the corresponding agreement to 10% change categories in PAC cardiac output measures. For example, at the 5% x-axis mark, the dashed red line represents the agreement in meaningful change categories between the cardiac index and the corrected flow time, where corrected flow time data has been dichotomized into three categories: 1. increased (by +5%); 2. decreased (by +5%); or 3. no meaningful change (pre- to post-PLR change < 5%), and cardiac index data has been dichotomized into three similar categories: 1. increased (by +10%); 2. decreased (by +10%); or 3. no meaningful change (pre- to post-PLR change < 10%).

Lastly, we assessed for agreement between any categorial changes in carotid US measures and PAC cardiac output measures (−10%, +10%, or no meaningful change). We did not observe significant agreement between any dichotomized changes in these measures (all *p* > 0.10 for weighted kappa, Figure 4) for either assessment interval. Although we observed increases in agreement for different values for fluid responsiveness compared to 10%, we did not find a value that resulted in statistically significant agreement between a carotid measure and a PAC cardiac output measure (Figure 4). Moreover, all possible cutoff values in carotid measures from 1 to 20% showed low agreement (all kappa < 0.25), with meaningful change (increases/decreases of +10%) in cardiac measures.

## 4. Discussion

In this study of 50 post-cardiac surgery patients, we did not detect a significant correlation between measurements of carotid blood flow and carotid blood flow time before and after a passive leg raise, when compared to Swan Ganz measurements. To our knowledge, this is the first study to compare pulmonary artery catheter (PAC) measurements with carotid Doppler ultrasound for assessing fluid responsiveness. While the PAC remains the clinical reference standard, it carries a reported 10% error when compared to the Fick method and serves as a surrogate in the absence of a true gold standard.

Previous studies have shown good correlation between carotid Doppler and noninvasive monitors like Cheetah-NICOM. However, our study did not replicate this correlation with the PAC, likely due to the inherent variability in both measurement methods. Bland–Altman analysis, commonly used to assess limits of agreement (LOAs), allows for an error margin of up to 2 standard deviations from the mean (±30% error), which may further contribute to these discrepancies.

Our study population consisted exclusively of post-cardiac surgery patients, which differs from previous studies that have often included mixed ICU cohorts, including septic and trauma patients. These populations may exhibit different hemodynamic responses and vascular compliance, potentially affecting the correlation between measurement modalities.

Assessment for fluid responsiveness through a passive leg raise maneuver has emerged as a reliable and dynamic method for guiding fluid resuscitation in hemodynamically unstable and critically ill patients [2]. Various invasive and noninvasive monitors have been employed for these measurements, and ultrasound has emerged as an excellent bedside tool, readily available for direct and repeated assessments. Ultrasound evaluation of the common carotid artery has been suggested as an easily accessible site for obtaining measurements to assess volume responsiveness during resuscitation [18].

Numerous studies have explored the feasibility and reliability of carotid (Doppler) ultrasound in predicting fluid responsiveness. Gassner et al. investigated the feasibility and accuracy of common carotid artery point-of-care ultrasound in measuring cardiac output compared to invasive methods, such as pulmonary artery catheter and pulse contour analysis, demonstrating an excellent interclass correlation coefficient [11]. On the other hand, Girotto et al. were unable to detect a hemodynamic effect, with an increase of less than 10% in cardiac index from baseline, after instantaneously measuring carotid Doppler blood flow during a passive leg raise [19].

ROC analysis in a study by Barjaktarevic et al., focusing on undifferentiated shock patients, found that a change in corrected flow time (ΔFTc) of 7 msec after a passive leg raise had a 97% positive predictive value and 82% accuracy in detecting fluid responsiveness using the Noninvasive Cardiac Output Monitor (NICOM). The average ΔFTc after passive leg raise for fluid responders was (14.1 +/− 18.7) msec, compared to 4.0 +/− 8 msec for non-responders [14].

Additionally, Ma et al. studied the correlation of carotid blood flow and corrected flow time (FTc) with cardiac output measured by thermodilution (PAC). They found that carotid blood flow exhibited a stronger and more consistent correlation with cardiac output measured by thermodilution and was less susceptible to measurement issues than (FTc) [13].

In a systematic review by Lance, published in the *Journal of Ultrasound Medicine* in 2020, assessing the accuracy of carotid ultrasound measures in determining fluid responsiveness in adults, the most supported measure was a change in peak velocity with respiration (ΔCDPV). Cutoffs ranging from 9% to 14% after a fluid challenge were identified. Although corrected flow time showed promise in the review, the heterogeneity in how this value is measured prevented the establishment of an optimal cutoff [19].

Joris van Houte et al. found a moderate correlation between carotid artery-derived cardiac output and contour analysis, with Bland–Altman limits of agreement of ±2.29 and ±2.57 L/min, biases of 0.1 and –0.54 L/min, and mean errors of 50% and 48% [20].

Maria C A-Granados et al. studied the correlation of carotid Doppler ultrasound with invasive cardiac output measurements (pulmonary artery catheter, PICCO catheter, and EV 1000 pulse contour analysis system) and found no significant correlation between carotid artery variables and invasive cardiac output measurements [21].

Among carotid Doppler measurements used to assess volume status, Joris van Houte et al. evaluated corrected flow time (FTc) for tracking cardiac output and stroke volume compared to invasive methods in cardiac surgery patients. FTc, calculated using Bazett’s equation, reliably tracked changes in cardiac output over time, although its ability to trend stroke volume was poor [22].

A recent meta-analysis of 13 trials evaluating carotid ultrasound for predicting fluid responsiveness in mechanically ventilated patients found that changes in carotid peak velocity (∆CDPV) demonstrated moderate diagnostic accuracy, with a sensitivity of 0.79 (95% CI: 0.74–0.84) and specificity of 0.85 (95% CI: 0.76–0.90) [23]. 

While other studies have previously explored the assessment of volume status and the measurement of cardiac output by using carotid artery POCUS, ours has some distinct advantages. Firstly, our study subjects were elective cardiac surgery cases with a thorough preoperative workup. This allowed us to exclude those with known carotid disease or a history of carotid surgery; our institution’s practice is to obtain dedicated carotid ultrasounds before cardiac surgery when indicated. We also compared our carotid artery POCUS cardiac output measurements, corrected carotid flow time, and change in ultrasound peak carotid pulsed wave Doppler velocity to cardiac output for fluid responsiveness measured by thermodilution, which is the gold standard for cardiac output measurement. Furthermore, we sought to improve result reliability by obtaining averages of three measurements at a time, as well as minimize inter-reader variability by having a limited number of intensivists trained in ultrasonography acquire, read, and perform measurements on the echo images. In addition, we tested correlations from a 1–10% change in cardiac output and performed a sub-analysis in patients who had a 10% increase in thermodilution cardiac output, without showing correlation with carotid artery ultrasound measurements. Increases in carotid corrected flow time (FTc) and change in peak velocity with respiration (ΔCDPV) in fluid-responsive patients were not reproducible measurements in our study, as in other studies, for unknown reasons. We hypothesize that our results may differ due to the possibility of different physiology in early post-cardiac surgery patients.

Our study has a number of limitations. These include the fact that this was a single-center study, which limits generalizability. Although we enrolled 50 patients, a larger sample size would be better powered to detect any associations between variables.

## 5. Conclusions

In postcardiac surgery patients, we were unable to detect a meaningful association between changes in carotid blood flow, corrected carotid flow time, or peak carotid systolic velocity variation (CDPV) and changes in cardiac output, as measured by PAC, after a passive leg raise that served as a surrogate fluid bolus.

Future research may be needed to validate the use of POCUS to determine fluid responsiveness in this patient population.

Our results suggest that POCUS of the carotid artery may not be an accurate method for assessing fluid responsiveness in postoperative cardiac surgery patients.

## Figures and Tables

**Table 1:** Description of Study Sample.

	(n)	(%)
Males	38	76
Hypertension	29	58
Abnormal HDL	25	50
Coronary Artery Disease	18	36
Diabetes Mellitus	29	16
**Total**	**50**	

**Table 2:** Associations between change in carotid measures and change in PAC cardiac output measures from pre- to post-passive leg raise. Results are reported as standardized betas from linear regressions with corresponding 95% confidence intervals. **Model 1** reports a crude association while **Model 2** reports an association adjusted for sex, body surface area, and disease history.

Variable	Initial Assessment β (95% Confidence Interval)		One-hour Assessment β (95% Confidence Interval)	
	Cardiac Output	Cardiac Index	Cardiac Output	Cardiac Index
*Model 1 (Unadjusted)*				
**Carotid Blood Flow**	0.08 (−0.22, 0.38)	−0.08 (−0.39, 0.22)	0.02 (−0.29, 0.32)	0.04 (−0.27, 0.35)
**Corrected Flow Time**	0.09 (−0.22, 0.39)	0.10 (0.20, 0.40)	0.02 (−0.28, 0.33)	0.01 (0.29, 0.32)
**Respiratory Peak Variation**	−0.18 (−0.48, 0.11)	−0.18 (−0.48, 0.12)	0.24 (−0.06, 0.54)	0.18 (−0.12, 0.48)
*Model 2 (Adjusted)*				
**Carotid Blood Flow**	0.12 (−0.22, 0.46)	−0.05 (−0.38, 0.29)	−0.15 (−0.44, 0.14)	−0.10 (−0.41, 0.21)
**Corrected Flow Time**	0.13 (−0.19, 0.46)	0.14 (−0.17, 0.46)	0.06 (−0.23, 0.35)	0.06 (−0.24, 0.37)
**Respiratory Peak Variation**	−0.18 (−0.51, 0.15)	−0.15 (−0.47, 0.17)	0.14 (−0.16, 0.46)	0.06 (−0.26, 0.38)

## Data Availability

The original contributions presented in this study are included in the article. Further inquiries can be directed to the corresponding authors.

## Abbreviations

The following abbreviations are used in this manuscript:

CVP	Central venous pressure
PAC	Pulmonary artery catheter
CO	Cardiac output
CI	Cardiac index
FTc	Corrected flow time
POCUS	Point-of-care-ultrasound
ΔCDPV	Change in respiratory peak systolic velocity
PWD	Pulsed wave Doppler
LVOT	Left ventricular outflow tract
TVI	Time velocity integral
CBF	Carotid blood flow
